# Between Multiple Identities and Values: Professionals’ Identity Conflicts in Ethically Charged Situations

**DOI:** 10.3389/fpsyg.2022.813835

**Published:** 2022-04-20

**Authors:** Lara Carminati, YingFei Gao Héliot

**Affiliations:** ^1^Faculty of Behavioral, Management and Social Sciences, University of Twente, Enschede, Netherlands; ^2^Surrey Business School, University of Surrey, Guildford, United Kingdom

**Keywords:** identity conflict dynamics, interpersonal and intrapersonal processes, behavioral and psychological responses, emotions, identity values

## Abstract

This study explored identity conflict dynamics in interpersonal interactions in professionals facing ethically charged situations. Through semi-structured interviews (*N* = 47), we conducted a qualitative study among doctors and nurses working for the English National Healthcare Service and analyzed the data with grounded theory approaches. Our findings reveal that identity conflict is triggered by three micro processes, namely cognitive and emotional perspective taking, as well as identifying with the other. In these processes, identity conflict is signaled by emotions and recognized as a clash not only *between* identities and their values, but also *within* one identity and its multiple values. Behavioral and psychological outcomes of identity conflict involve seeking peer support, doing reflective practices and identity growth. This article contributes to identity literature by providing a multilevel approach of identity conflict dynamics able to account for both interpersonal and intrapsychic processes, deeply hold values and emotions, as well as crucial behavioral and psychological consequences.

## Introduction

Due to the more complex and diverse nature of today’s society, professionals are increasingly asked to juggle different identities at their workplaces (Wright et al., 2017). Even though these identities guide people’s attitudes and behaviors, given their dynamic nature (Ashforth and Schinoff, 2016), enacting and managing multiple identities can be challenging (Ahuja et al., 2019). Consequently, when identities are triggered simultaneously, but are incongruent, identity conflict may arise (Brown, 2017).

Identity conflict is defined as a sense of discrepancy between the beliefs, norms and expectations held by an individual (Horton et al., 2014). Studies have provided strong theoretical and empirical bases for understanding the optimal strategies to solve identity conflict, ranging from identity integration or assimilation to identity separation or compartmentalization, or even identity annihilation (Kreiner et al., 2006; Petriglieri, 2011; Ramarajan and Reid, 2013). Research has also explored boundary conditions that can ameliorate individuals’ perception of identity conflict. For instance, Héliot et al. (2020) have identified psychological safety as a key factor in reducing potential identity conflicts and therefore contributing to individuals’ wellbeing and effectiveness. Nonetheless, this research has generally overlooked the unraveling of identity conflict dynamics in interpersonal interactions (Ashforth and Schinoff, 2016; Islam, 2020; Ramarajan and Reid, 2020). Given the vital relational component that is intrinsic in individuals’ identities to provide meanings to the self (Brewer and Gardner, 1996), understanding individuals’ perception of identity conflict in such interactions becomes pivotal in order to elucidate identity dynamics (Caza et al., 2018).

Yet, research on identity and identity conflict has mostly focused on identities in their entirety, as ‘monolithic entities,’ without doing a more profound exploration of the role played by those deeply held values and associated emotions that constitute identities and can initiate processes of identity conflict (Wright et al., 2017; Ahuja et al., 2019). Values are the pillars from which individuals define who they are and how they should act (Schwartz, 2016). They are at the heart of many professions and are strongly linked to emotions, since individuals who value their identities, and the meanings associated with them, also have an emotional investment and attachment to such values and identities (Wright et al., 2017). Hence, since identities are “value-soaked” (Ashforth and Schinoff, 2016, p. 122), unpacking the role that values and emotions play in identity conflict dynamics becomes paramount to understanding individuals’ behaviors. Nevertheless, with just few notable exceptions (Croft et al., 2015; Wright et al., 2017), scholars have not explored identity conflict at a deeper cognitive and emotional level and its link with individuals’ actions, or barely investigated how individuals respond behaviorally when threatened by identity conflict (Petriglieri, 2011; Ramarajan and Reid, 2013). Little is still understood about how professionals navigate the tensions around their values and emotions when experiencing identity conflict in interpersonal interactions and the consequences of such conflict on their behavior.

Thus, we set out to discern these dynamics within the medical context through an inductive, qualitative study of healthcare professionals working for the National Healthcare Service (NHS) in England. We paid attention to how such professionals perceive, and respond to, identity conflict and moral struggles when interacting with their patients in ethically charged situations^1^. In broad terms, our findings suggest that professionals experience identity conflict as being triggered by interpersonal micro processes that can activate latent, contrasting values and emotions, and that they respond by actively seeking support from peers or through reflective practices. Surprisingly, in our results conflict is generally perceived as a positive contribution to personal growth. This study thus unpacks the deeper components of identity conflict and describes how individuals engage in a series of interpersonal dynamics that can shape individuals’ subsequent behaviors. We also suggest new directions for identity and decision-making literatures, as well as insights for professionals and organizations into how to reduce individuals’ moral struggles.

More specifically, we make three key contributions. Firstly, we extend current identity literature by considering rarely studied contextual and interpersonal dynamics when exploring intrapsychic identity conflict in professionals (Ashforth and Schinoff, 2016; Ramarajan et al., 2017; Caza et al., 2018; Carminati and Gao Héliot, 2021). This multilevel perspective (Islam, 2020; Carminati and Gao Héliot, 2021) allows us to better explain, and grasp, the equilibrium between the self and the surrounding environment, especially when the latter may function as a trigger of identity conflict. Secondly, we advance identity research by unraveling how values, together with emotions, are linked to identity dynamics and can influence the perception and responses to identity conflict (Wright et al., 2017; Ahuja et al., 2019; Huhtala et al., 2020). Lastly, we extend pivotal theoretical studies on identity conflict consequences (Petriglieri, 2011; Ramarajan and Reid, 2013) by further identifying behavioral responses that individuals may implement when experiencing identity conflict in ethically charged circumstances, as well as novel psychological consequences characterized by positive connotations.

The remainder of the article is structured as follows. After providing a brief review of the current identity approaches and virtue ethics perspectives in relation to professionals and ethically charged circumstances, we detail our data collection and analysis methods, followed by the main results of our investigation. Lastly, we discuss our findings and conclude the article by highlighting its limitations, directions for future research and practical implications.

## Theoretical Background

### Identity and Identity Conflict

Recent identity approaches have underlined that identity conflict reflects a discrepancy between “values, beliefs, norms and demands inherent in individual and group identities” (Horton et al., 2014, p. 6) and emerges when individuals feel they must give precedence to one set of meanings and behaviors over another to satisfy particular identity-based expectations (Ramarajan, 2014). Even though identity conflict is mostly an intrapersonal experience (Ramarajan et al., 2017), identity conflict dynamics do not happen in a vacuum and deserve to be explored in more complex, real-world scenarios. Such a real-world exploration will enhance our understanding of how identities are dynamically co-constructed, revised and re-arrange in relation to often neglected contextual triggers (Alvesson et al., 2008; Caza et al., 2018; Ramarajan and Reid, 2020). Indeed, a vital aspect of self-meanings are individuals’ relational identities or individuals’ self-understanding in relation to others (Brewer and Gardner, 1996; Sluss and Ashforth, 2007; Islam, 2020). Hence, this article specifically focuses on how interpersonal interactions can trigger intrapsychic identity conflicts, i.e., those conflicts that unravel within individuals’ mind and self.

### Values, Emotions and Behaviors

In such intrapsychic conflicts, values play a salient role. Values have been defined as guiding principles, motivators and the foundation of people’s attitudes and behaviors in organizations (Schwartz, 2016), especially in professionals. Professionals possess unique knowledge and skills that can benefit and bring value to society (Macdonald, 1995). Values, such as commitment, fairness and altruism, go beyond self-interest, power and privileges and represent the key moral drivers for professionals’ identities and actions (Wright et al., 2017; Huhtala et al., 2020). Since professionals are subject to external demands and societal expectations (Ramarajan and Reid, 2020), they rely on values –and their associated actions–to create and express a sense of being competent when enacting a specific work identity (Caza et al., 2018). Still, as if identities were monolithic entities, scholars have paid limited attention to investigating identity at a deeper level and have missed the importance of values in influencing professionals’ identity conflicts, behaviors and interactions at the workplaces. This holds particularly true when considering that the same values have the potential of being interpreted differently (Wright et al., 2017), hence influencing identification processes and creating a kaleidoscope of remarkably different outcomes.

To understand how values can impact these processes, we integrated recent identity theory approaches and identity conflict literature (Ramarajan, 2014; Ramarajan et al., 2017) with virtue-based ethical approaches from the decision-making literature. In these person-centered approaches, individuals’ behaviors reflect the virtues guiding people’s choices of action and become indicative of individuals’ moral character (Uhlmann and Zhu, 2014; Uhlmann et al., 2015). Indeed, building on the fact that individuals’ moral behaviors are driven by universal values representing ethical principles (Kohlberg, 1969), research on ethical decision making has provided an alternative lens for the vital role of values in individuals’ choices and in reflecting those identities that individuals have prioritized in ethical dilemma situations. Ethical dilemmas are defined as conflicts between two possible moral imperatives, neither of which is unambiguously preferable nor in which obeying one would result in transgressing another (Kidder, 1995). Given this definition and the fact that all values are fundamentally moral (Frankena, 1973), we associate identity conflict to ethical dilemmas and adopt a value-oriented perspective on identity. Going beyond course-grained conceptualization of identities, this perspective helps us to extend our current knowledge of identity conflict by unpacking intrapsychic identity dynamics and accounting for nuances – e.g., in relation to emotions – that could be otherwise lost without a filigree approach. Hence, by focusing on the values constituting individuals’ multiple identities, virtue-ethics approaches could help to address how individuals perceive identity conflict and respond to it.

Devoting attention to the role of values in identity dynamics also brings interest in the part played by emotions (Schwartz, 2016). Research has underlined that emotions are strictly connected to professionals’ values because people who are “committed to the values of an institution [profession] really care and […] have high cognitive and emotional investment in the institution [professional] order” (Wright et al., 2017, p. 204). Since professionals’ values are interlaced with others’ best interests, then whether an action is perceived as right or wrong may generate morally inclined emotions (Haidt, 2003). Recent studies have started exploring the constitutive power of emotions in identity construction (Cascón-Pereira and Hallier, 2012; Croft et al., 2015; Ahuja et al., 2019) and there is growing acknowledgment that emotions can affect the processes and outcomes of identity work (Caza et al., 2018; Winkler, 2018). However, little is still known about the role of emotions, especially morally driven ones, in relation to values and identity conflict.

Similarly, scarce research has explored behavioral consequences of identity conflict dynamics. Theoretical framework and empirical studies have dedicated most of their attention to the psychological outcomes of identity conflict experiences (Ramarajan and Reid, 2013; Karelaia and Guillén, 2014; Carminati and Gao Héliot, 2021). As to what people actually do and how they react in response to it has had less investigating (Caza et al., 2018). Since individuals’ behaviors are driven by their identities, values and emotions (Brown, 2017), what people do can significantly reflect what happens in their minds and is felt in their hearts. This is especially true when people are struggling within themselves to be both professionally effective and conscientiously moral at the same time (Bardon et al., 2017; Huhtala et al., 2020). Hence, to understand individuals’ behavior in a specific situation, it is important to trace back and link the action to individuals’ values and emotions, and thus explore the identity conflict-behavior relationship.

To sum up, the overarching goal of our study is to investigate interpersonal and intrapsychic dynamics of identity conflict, as well as to understand its consequences on professionals’ behaviors. We aim to achieve this by developing initial answers to the following research questions: (i) How is identity conflict perceived in interpersonal interactions and experienced at a deeper level in ethically charged situations?; and (ii) What are the potential behavioral responses to identity conflict in ethically charged situations?

## Research Methodology

### Research Design

We conducted an inductive, qualitative study using grounded theory approaches (Strauss and Corbin, 1990) for two reasons: (i) grounded theory approaches are often opted for when the researchers’ focus is on human behavior patterns and key participants’ actions, as well as contextual and processual elements surrounding the experience under examination (Suddaby, 2006); (ii) since their purpose is to develop theoretical insights into how individuals understand and make sense of reality subjectively, they are appropriate for studying a deeply subjective phenomenon like identity conflict (Suddaby, 2006). Through an interpretive discursive lens (Miles and Huberman, 1994), our purpose was to produce a rich account of, and explore, participants’ voices, as well as interpret and give meaning to their narratives in order to extend current theories from the spontaneous emergence of novel themes (Strauss and Corbin, 1990).

### Justification of Sampling and Context

We investigated how healthcare professionals, namely doctors and nurses, working for the English NHS perceive and react to identity conflict and moral struggles in interpersonal interactions when facing ethically charged situations. Although doctors and nurses have specific and different medical identities that are associated with their medical training and education, they also share a common medical identity related to their belonging to the healthcare workforce. This healthcare population has both unique and generalizable characteristics, making it a peculiar one to study.

Healthcare professionals are not only considered as working in a prototypical profession (Strauss, 1975; Pratt et al., 2006), but are also constantly involved in interactions with patients since the doctor/nurse-patient relationship is a core element of the ethical and professional principles they abide by Diamond-Brown (2016). Indeed, whilst doctors and nurses’ professional identity is grounded in a codified system of values and principles by which they should abide as part of their profession (Ruitenberg, 2016), the application of these principles is not always straightforward, especially when dealing with patients’ lives (Birchley, 2012). Consequently, their personal and professional values, together with their emotions (e.g., empathy, compassion, etc.), are often heavily questioned during the daily clinical and medical activities, leading to moral impasses and ethical dilemmas (Borgstrom et al., 2010; Genuis and Lipp, 2013; Kälvemark et al., 2004). Therefore, compared to broader workforces, healthcare professionals involved in ethically charged situations face stronger ethical identity conflict, because of the sensitive and moral nature of their job (Borgstrom et al., 2010).

However, contributing to making identity conflict dynamics more generalizable (Eisenhardt, 1989), like many other professionals (e.g., lawyers or politicians), doctors and nurses’ work is in the eye of the public, which can accentuate the intensity of their identity conflict even more (Kreiner et al., 2006), especially in ethically charged situations. They also engage in various extra activities, ranging from managing hospital resources, treatments costs and making decisions about quality of life (Genuis and Lipp, 2013), which may further put pressure on them. Additionally, as in other professions, in the healthcare profession there are multiple layers of specialization, differentiation and qualification levels. Therefore, we made sure that our sample was remarkably diversified to reflect the complexity of this professional world. We purposively selected 12 hospitals for their characteristics, such as the services provided (e.g., palliative care, oncology, gynecology, and intensive care), size (small, medium and large hospitals providing the services we were interested in), personnel (e.g., junior and senior consultants, specialist nurses, matrons, and senior nurses) and location (to cover the breadth of the National Healthcare Service within England). These characteristics help to add both in-depth descriptions and generalizability. Hence, doctors and nurses represent an exemplary profession that can significantly experience identity conflict between their professional and personal values at the workplace, especially in ethically charged situations.

These situations have become increasingly frequent and challenging in today’s society. They represent one of the most common circumstances preceding death in Western countries (Rietjens et al., 2012) and more than 50% of European and American doctors perceived ethical struggles in these situations (Hurst et al., 2005, 2007). Hence, ethically charged circumstances can exacerbate the dynamics of identity conflict making them more observable. These characteristics make ethically charged situations suitable for representing an extreme and unique context (Eisenhardt, 1989). Indeed, changes in social mores over the 20th Century have given rise to uncertainty and created room for subjective interpretations of the course of actions in ethical collision situations (Genuis and Lipp, 2013). Although some constitutive principles of medical ethics exist to guide healthcare professionals in their duties, responsibilities and conduct (Birchley, 2012), as to *how* healthcare professionals should act to avoid moral impasses is a delicate matter (Borgstrom et al., 2010; Ruitenberg, 2016).

As exemplified by the COVID-19 pandemic, ethically charged situations can make doctors and nurses’ professional identities and values quiver (Segers, 2020; Chirico et al., 2021). Horton et al. (2014) have noted that change events can be key triggers of identity conflict since they may invoke multiple identities with different values and role-based expectations. Similarly, we focused on ethically charged situations since in these circumstances healthcare professionals call upon their multiple but potentially contrasting personal and professional values, regarding for instance what constitutes quality of life or human dignity, and which emotions to display in each situation (Ruitenberg, 2016). The emerging identity conflict can impact healthcare professionals’ psychological outcomes (Genuis and Lipp, 2013) and decision making (Hurst et al., 2005), as well as patient care and the quality of the healthcare system (Kälvemark et al., 2004). Consequently, ethically charged are possibly the most complex circumstances that healthcare professionals may encounter. Nonetheless, to pursue the correct action, an identity conflict of moral nature must be faced and resolved. Hence, ethically charged situations will enable more transparent observations of healthcare professionals’ perception of, and responses to, identity conflict (Eisenhardt, 1989). Therefore, our sample and context offered a unique opportunity to explore both how identity conflict is perceived in interpersonal interactions and behavioral responses to identity conflict in ethically charged situations.

### Research Instruments

We interviewed a total of 50 healthcare professionals (21 doctors, 11 = F and 10 = M, and 29 nurses, 25 = F and 4 = M) during the period March-June 2018. The first 3 interviews were conducted as a pilot and helped the first author to refine the interview protocol for the other 47 main in-depth interviews. Ethical approval to conduct the research was obtained from the University Ethics Committee and the Health Research Authority, and data were safely stored on the University computer, protected by a password. The interviews were semi-structured and conducted face to face (*N* = 43) or *via* video conferencing (*N* = 4) by the first author at the participants’ workplaces. We chose semi-structured interviews since they were deemed appropriate to probe more deeply into identity conflict perception and they are particularly effective at uncovering people’s sense of self and identity (Miles and Huberman, 1994). They were audio recorded and transcribed verbatim, concealing the participants’ identity and personal information. Names used in this article are pseudo names. The interviews, on average 60 min in length, followed a standard set of 20 questions (see Supplementary Appendix A for a selection of the interview protocol) to facilitate the identification and comparison across the emerging themes (Miles and Huberman, 1994) and were formulated following organizational and medical studies on the topic (Hurst et al., 2005; Kreiner et al., 2006).

The doctors and nurses were asked about: (a) the motivation behind their decision to become a doctor or a nurse in their specialty; (b) the various professional and personal values associated with their multiple identities and roles; (c) their perception of a personal struggle, conflict or ethical dilemma they experienced in their clinical practice; (d) the reasons why they deemed struggle/dilemma to be particularly important to them and the role played by the multiple identity values they associated with; (e) their views regarding Baby Charlie’s case, a recent and dramatic ethical dilemma in the United Kingdom involving an infant with a genetic and degenerative disease; and (f) what they did when experiencing their conflict at work.

### Data Analysis

We analyzed the data iteratively, going back and forth between our data, and an emerging structure of themes and theoretical arguments (Strauss and Corbin, 1990; Miles and Huberman, 1994). This process entailed a constant comparison and recoding of previously coded texts with new coded sentences, as well as looking for further evidence in our data any time a new conceptual category emerged. Hence, it involved discarding some schemes, collapsing similar codes, and varying others. During this phase, we had regular and long discussions regarding our interpretations and structure of the data and codes. We relied strictly on the language used by the participants to frame issues and concepts in our findings to maintain a connection with the raw data. We also read up on a broad range of topics, from identity values to emotions, and from ethical decision making to interpersonal dynamics, to understand in a mutual process how existing research could help us refine our emerging insights from the data, and how these insights could, in turn, contribute to the existing literature. After coding 38 interviews we found no additional new codes in the remaining 9 transcripts. The absence of new codes gave us confidence that we had reached saturation, the point at which further data would yield redundant responses and confirm existing insights (Strauss and Corbin, 1990).

We also conducted case-comparisons of codes between doctors and nurses to understand whether and how these two professions varied systematically in their perception of identity conflict. Overall, our exploration of the cases revealed more similarities than differences in terms of their experience of ethical struggles. This may be due to the fact that the interviewed nurses had great autonomy and independence in their decision-making capacity. It is also worth noting that all the participants experienced identity conflict in terms of clashing values and emotions. However, our analysis also revealed some variations in the behavioral responses associated with identity conflict: whilst both doctors and nurses tended to rely equally on their peers for advice and support, nurses were the ones that engaged the most in reflective practices. Nonetheless, all the healthcare professionals moved through the same processes of identification with the other (i.e., the patient) in terms of triggers of identity conflict. Therefore, we built a new framework based on healthcare professionals’ commonalities in identity conflict dynamics.

**FIGURE 1:**
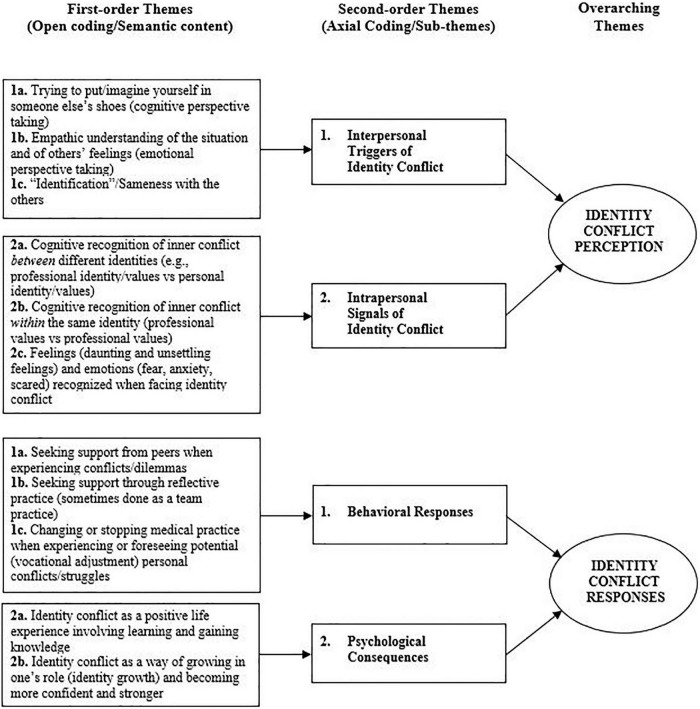
Data structure.

Regarding our first research question, i.e., how identity conflict is perceived in interpersonal interactions and experienced at a deeper level in ethically charged situations, we established second-order themes that captured both the intrapersonal perception of identity conflict and the interpersonal dynamics leading to such conflict perception. Concerning our second research question, i.e., what are individuals’ behavioral responses to identity conflict in ethically charged situations, we included first-order themes, namely ‘seeking support from peers and through reflective practices,’ ‘stop/change in career,’ as well as an interesting second-order theme related to the positive psychological consequences of facing identity conflict in the long term. Figure 1 summarizes our data analysis and the themes that emerged. The NVivo11 software program was used to organize the data and for the analysis.

## Findings

We present our findings in relation to each research question. To gain maximum parsimony, we interlace both the first order and second order findings throughout this section to provide a well-grounded structure and an in-depth description of our data. Supplementary Appendix B Tables 1, 2 provide further illustrative quotes and how they relate to the first-order themes. We begin by presenting the findings related to the first research question.

### Healthcare Professionals’ Perception of Identity Conflict

Two key themes were developed for our first research question from the participants’ dialog about their perception of identity conflict in the workplace, namely ‘Interpersonal triggers of identity conflict’ and ‘Intrapersonal signals of identity conflict.’ The first theme illustrates the three interpersonal processes responsible for instigating identity conflict in the participants. The second theme unpacks the cognitive and emotional recognitions able to alert the healthcare professionals that an inner identity conflict was about to happen.

#### Interpersonal Triggers of Identity Conflict

All the participants spoke passionately about the numerous values – sometimes also referred to as “attributes” or “principles” in the medical literature (Halligan, 2008; Abbasinia et al., 2020; Birchley, 2021) – driving and supporting their professions, from pursuing ‘patient best interest’ to ‘being their advocate’ and ‘establishing a connection with them.’ Since it was crucial for them to respect these values as they were associated with the interactional dynamics that constitute their professional-patient relationship, the first derived micro process was the cognitive ability to take the other’s perspective. Establishing a connection and a first point of contact with their patients facilitated the fulfilment of the participants’ professional roles. As this nurse highlighted:

I think that, personally and professionally, it’s vital to recognize how things can be seen from other people’s perspectives […] you need to put yourself in other people’s shoes to really understand your patient and their family…and do your job, really. (Sarah, Nurse, Female)

The participants balanced this process of putting themselves in their patients’ shoes carefully because ‘you need to translate “what would be ok for me?” into “what is good for my patient?”, since what might work for me, might not work for somebody else.’ (Tony, Nurse, Male).’ Trying to maintain this fine balance entailed exercising a strong psychological control over personal beliefs and principles.

Such cognitive connection with patients was also associated with a second micro process, namely developing an empathic understanding of, and concern for, their patients. The ability to understand others’ feelings was described by the participants as a pillar of the healthcare professional-patient relationship because it enabled one to understand better what the patients were experiencing through compassion, sympathy and empathy. As this nurse noted:

I could understand, I could feel what she was going through…and I couldn’t help putting myself in her situation and thinking, if I were in that situation what decisions would I make? […] and I think we all do that, you can’t stop doing that, you can’t stop feeling that way…it was so emotional. (Rachel, Nurse, Female)

However, this powerful cognitive and emotional process of understanding others’ thoughts and emotions through perspective taking was a double-edged sword. On the one hand, imagining other people’s thoughts and feelings helped the healthcare professionals to recognize what was important for their patient and how to meet their expectations. On the other hand, by clouding any rational and objective decision making, it could make their professional judgment waver, and an already ethically charged decision making situation even harder. The fine equilibrium between professional and personal values and emotions was difficult to sustain, especially in ethically charged situations, due to the high cognitive and emotional demands placed upon healthcare professionals. The participants acknowledged that, in critical situations, getting emotionally closer to patients was inevitable and this, together with influences from personal values, could lead to experiencing conflict. As if trying to balance the instability of her identities by asserting a distinction between her “selves” (i.e., “I,” “me,” and “my”) and her patient (Harding et al., 2014), an oncology doctor described:

I could understand her worries and it really made me cry…it struck a chord […] I think I probably struggled with my decision because I could see her point of view on saying ‘I want more treatment’…but then, because of my experience, and knowing how this disease goes and how she would probably feel worse with the treatment, I knew it would be deleterious for her…but how could I say ‘no’?. (Mary, Doctor, Female)

Similarly, on recalling a dramatic event involving a drunken mum causing a car accident in which her little son died, this other nurse noted:

I felt for her…I could really feel for her […] so the moral conflict for me was that I knew that when she left that resus room, she would be arrested and would never see her little boy again […] but I knew that, at some point, I had to say, and I did say, ‘we need to go, I need to take you outside’. (Becky, Nurse, Female)

Thus, cognitive and emotional perspective taking was perceived as something necessary for providing high-quality care, but also as interfering with clinical decision making. This paradoxical nature of perspective taking processes grabbed our attention because they seemed to exacerbate the identity conflict experience, by activating the moral values and emotions that are constitutive of those identities the participants had in common with their patients. Indeed, sharing values and emotions could facilitate a third micro process of identification with the other, i.e., the patient. It was clear from the participants’ words that there was an interpersonal perception of identity conflict due to a sense of ‘sameness’ functioning as a trigger. By identifying with their patients, the participants’ professional values and identities were hindered by the more personal values, emotions and identities stimulated in that specific circumstance. Such an interpersonal process was depicted well by this doctor in palliative care:

It’s seeing people who are in very similar situations to ourselves…so, I’m a young mother and I struggle when I look after a young mum, because it is too much like my own situation […] I truly think that the danger for me really comes when you start to draw parallels between somebody else’s situation and your own. (Jess, Doctor, Female)

The fact that the participants could connect strongly with the person in front of them stimulated doubts, reconsiderations and uncertainty about the way they should have acted compared to what they would have done if they had truly followed their medical identity. For instance:

Last year I was looking after a chap who was my age, he was a medic, he was married with two young children under the age of ten like mine […] I think that this experience particularly stayed with me because there was that kind of strong connection…you know, you relate to that family, because they are a similar sort of people, similar upbringing, similar background […] So, I think the difficult bit for me was probably my ability to relate to his position…I could see myself lying in that bed and thinking ‘What would I want someone to do in that scenario?’ […] I am not ashamed, I felt conflicted really…it was a terrible situation, there was nothing left. (Luke, Doctor, Male)

Emotions also played a crucial role in this process of identification because they functioned as the fastest route channeling the process. Transcending the social roles and creating insecure identity positions, emotions fueled the sense of similarity between the healthcare professionals and patients and stimulating the conflict. As this nurse said:

I think my conflict was that it felt personal…personal because of the patient age, because she was nearly the same age as me, which makes it a bit more real…and you can put yourself in that person’s position and in her family’s position, because you know how you would feel if it was a relative of yours…and I suppose that’s where the emotions come in, because, you know, this is just how you would feel…you would feel upset, particularly knowing what’s going to happen. (Emily, Nurse, Female)

Thus, the breaking point of the perfect balance between professional and personal values and emotions was a series of combined and interrelated micro processes unraveling at an interpersonal level. These interpersonal processes of perspective taking and identification were strongly interlaced and concerned the dynamics of values and emotions inherent in the healthcare profession and represent the ‘why’ the participants experienced the conflict. Indeed, the unfolding of such dynamics in ethically charged situations created a sense of misalignment from an ideal condition of alignment. The result is a conflict, struggle, or dilemma that the healthcare professionals perceived in their clinical practice. The following section now addresses how the participants experienced conflicting values and identities.

#### Intrapersonal Signals of Professional Identity Conflict

As just shown, identity conflict was triggered and initiated by processes of interpersonal interaction in the professional-patient relationship. These interpersonal experiences of identity conflict, spilling over to how the professionals position themselves in relation to their own identities and values, set in motion intrapersonal experiences along three dimensions (i.e., a cognitive recognition of inner conflict *between* identities, a cognitive recognition of inner conflict *within* identities and an emotional recognition of inner conflict), which function as signals of identity conflict.

Firstly, there was a cognitive recognition of the inner conflict related to a tension *between* their professional and personal identities and the moral values linked to them. The participants’ perception of struggles, uncertainty and indecisions was indeed linked to their inherent sense of morality. One of the most common conflicts was between their medical identity and their family identity, since the participants often called upon their values of being a mother, a father, a daughter or a son, to judge, make decisions or evaluate the challenging situations they were facing in terms of what was the *right* course of action. This nurse said, for instance:

Obviously, as a mother I have some values and those values tell you that you would fight for your children, wouldn’t you? But then looking at it from a nursing point of view, with my professional hat on, you would also agree with the doctors’ decision, because it’s about the child, not the family […] Still, I don’t know…It’s such a destroying situation, isn’t it?. (Anne, Nurse, Female)

Similarly, the participants’ religious identity was significantly involved in a conflict with their professional identity. As the quote below shows, the moral values underpinning the religious identity were the main reasons for the identity conflict experience since they were clashing and causing uneasiness within the participants’ professional identity:

Some areas of Christianity believe very strongly that you must always accept any treatment, push for every treatment, you have to prolong life as much as possible because it allows God to do a miracle and if you ‘give up’ then how can God to do that? […] but the trouble for me is…some Christians I’ve come across, even in a professional context, won’t even let themselves think about what might happen if this miracle doesn’t occur… that’s actually a real problem for me, because maybe you are putting somebody through something futile which, in itself, is unethical and unprofessional. (Jess, Doctor, Female)

These types of conflicts clearly reflect those identities and values that the healthcare professionals deemed salient and central to themselves and that were activated by the specific situation they were in. In this sense, the three micro processes highlighted before can trigger an identity conflict involving all the different identities considered in their entirety. However, we also pinpointed a second type of inner conflict.

This conflict unfolded *within* the participants’ professional identity and *between* its values. They stressed that medicine is both a science and an art, a ‘balance between the analytical aspect of the practice, where you come to a judgment in which you could have conflicting evidence, and the human side of the practice’ (Alex, Doctor, Male). Such an intrinsic nature of medicine implies that there is room for subjective interpretations of some medical principles (Birchley, 2012), which also means that medical principles can be ‘bent’ depending on the influence of other values, beliefs or emotions that can be associated with them. In ethically charged situations, some healthcare professionals may perceive some medical values as less binding, less straightforward, or even contrasting, leading to inner conflicts. For instance, this doctor in palliative care spoke about assisted suicide:

It’s a real dilemma, because as a medic you don’t want to let the patient down, because you can’t control their pain appropriately […] It’s a conflict in the sense that one side of me says that [assisted suicide] is the right thing, because the patient should have the autonomy to decide…but the other side of me says that, as a doctor, my values are to save lives…we are trained not to cause harm, that’s such a core principle for us, and that’s a big problem […] So, I don’t know, maybe it’s me, my morality, but it’s as if the whole concept of ‘do not harm’ has a double effect […] So, your values of being a doctor and saving lives conflict with your duty of care to the patient. (Matt, Doctor, Male)

In this case, the two medical values of the participant’s professional identity were perceived as clashing due to some associated moral values important to the participant, resulting in one specific medical principle rather than the other. Similarly, this doctor in oncology noted:

I suppose, a lot of the ethical decisions that often come with End-of-Life are related to ‘how far do you take the treatments’ or ‘when do you stop?’ ‘When is it time to stop investigating and stop doing things […]?’ So, I suppose the conflict for me is ‘How much do you do things? And ‘How much do you not do things?’ […] Because ‘is living a long but miserable life better than having a short but happy life?’ It should be up to the patient to decide, but sometimes it’s not always possible. (Fran, Doctor, Male)

What emerges from these quotes is a set of medical values and principles which coexist in, and belong to, the same professional identity, but are potentially connected to other values that are fundamentally moral and deal with the ‘rightness’ of doing an action. The *leitmotiv* here is thus the intrinsic moral nature of the values generating the identity conflict. Deciding which principles or values should be prioritized depended on the situation and the patient, as well as the cognitive and emotional demand involved, and can lead the individuals to experience a moral conflict within the same identity. In this sense, identities are multifaceted and polyhydric entities in which each value can play an independent and important role in triggering the conflict.

Lastly, the third recognition of identity conflict we identified was related to the participants’ emotions and their roles in ethically charged situations. Adding a more ‘visceral’ dimension and perspective to the notion of identity conflict, healthcare professionals described how they felt when they experienced the conflict. An oncology doctor noted:

I think you feel unsettled in yourself if you are battling with something…it’s something you can’t put to bed, it’s niggling there […] for the rest of the clinic I was a bit upset and thinking ‘what are you going to do? What are you going to do?’ in the back of my mind, and I couldn’t concentrate. (Katy, Doctor, Female)

As for values, these emotions were interlinked with the *right* course of action the participants aimed to pursue and, as such, could be considered as inherently moral. Indeed, the emotions and feelings the participants experienced reflected the potential behavioral responses they should prepare themselves to engage in. Here, the emotions acted like a signal for the individuals, that a tough cognitive decision and its subsequent behavior needed attention:

I’m probably a bit anxious, you know, as to what kinds of responses are meant to be made and how I would I respond to it [the conflict] …Would I be prepared? So, I’m probably scared not be ready enough to do what I think is the right thing to do […] I guess, I feel a certain level of uncertainty…anxious that you are getting it wrong and what the implications of what that will be, both ethically and legally…you want to please people, but sometimes it’s not that easy to go down that route, sometimes you can’t, you simply can’t…and so you feel frustrated and helpless. (Tom, Doctor, Male)

Morally entwined emotions were thus a constituent part of identity conflict. They warned individuals not only about the emergence of the conflict, indicating dissonance and discomfort, but also during the conflict about the potential outcomes and actions stemming from choosing a value over the others. In this sense, by going beyond the sense-making mechanisms, they guide the enactment and the establishment of the self that the individual has opted for.

### Healthcare Professionals’ Responses to Identity Conflict

Our second research question unraveled how healthcare professionals responded behaviorally to the perception of identity conflict in ethically charged situations. As illustrated in Figure 1, the healthcare professionals reported a series of behavioral responses, ranging from searching and receiving peer support, to reflecting about and changing their practice, as well as positive psychological consequences of identity conflict on their identity.

#### Behavioral Responses to Identity Conflict

Seeking peer support was the first theme the participants described in response to what they did when they perceived a conflict. This was particularly interesting because healthcare professionals are often associated with the idea of autonomous and ‘solo-person’ jobs. A doctor in palliative care said:

We do not work in isolation, it’s not just me, about myself […] so, if I felt there was something I just didn’t feel right about, and maybe I was struggling with, I would go to my peers and sit down and ask ‘can we reflect on this please?’…in my experience this is kind of the only way to approach dilemmas…to have a lot of different inputs, to come and talk about…[…] sometimes just identifying and voicing the conflict you are in can be really, really helpful for you and for everyone to know. (Birgit, Doctor, Female)

Similarly, this experience is shared by a nurse:

“I think what I probably find the best part of dilemmas is recognizing that it’s not a solo person job or responsibility…it is not down to me to solve, it’s not down to me to find a solution, and it’s very interesting to hear all the points of views of the other people in the team…and very advantageous to have someone else saying “hang on a minute, have you thought about that?” […] and when you evaluate it, you realize the benefits of being part of a team” (Miriam, Nurse, Female)

Hence, sharing personal struggles with the ‘peer fraternity or sorority,’ was a common practice that both the doctors and nurses pursued actively in order to find a solution to the dilemma or conflict they were experiencing especially since ‘there aren’t clear answers for a lot of the dilemmas we face, so you are very dependent on what the fraternity or sorority would do in a similar situation.’ (Hannah, Doctor, Female).

The second theme manifests the way the participants responded to identity conflict through reflective practice. The goal of this practice, which was sometimes done as group reflection and was especially common among nurses, was to improve clinical practices ‘with the idea of “would I have done anything differently?” and revaluate what you think is right or would do differently next time.’ (Eva, Nurse, Female). This holds especially true for ethically charged events. According to this doctor, moral conflicts are cases in medicine that, by nature, lead healthcare professionals to reflect on and conduct an introspective analysis:

You know, you go back on things when you’ve been affected, and those [conflicts] are certainly significant events, so are meant to affect you, one way or the other, probably not on the spot, so you probably think about what you have actually done, and ask ‘have I done the right thing? Have I not done the right thing?’…you may doubt a bit, but then, you know, you reflect…and reflecting is probably the way, or I find it’s my way of dealing with these events. (Will, Doctor, Male)

Echoing the same concept:

I suppose that what you learn from being a nurse is following a kind of reflective practice […] when I’m speaking to staff around death and dying, it [reflective practice] helps me to emphasize those learning experiences a bit more and it takes a lot of reflecting […] so, when things are more challenging, and they might go wrong, you kind of reflect with your team and hopefully learn from it. (Erwin, Nurse, Male)

Hence, when the participants perceived conflicts or dilemmas they could rely on the help of others, their peers, with whom they could either discuss the struggle they were experiencing or reflect on it later on, to be prepared for similar, future cases. Lastly, the participants responded to identity conflict through more drastic actions, such as making changes in, or stopping, their practice. Although this theme was not frequent, we found that the cases in which it did happen illustrated a remarkable behavioral response to identity conflict perception. For instance, this nurse decided to withdraw temporarily from the nursing profession due to a strong conflict she could not cope with:

The impact it [the conflict] had on me afterwards and the cost of it was difficult…I left nursing […] that was the point when I thought ‘I’m not ready yet, I need to get some life experience and do some other things and then come back to this’…and that’s what I have done. (Lauren, Nurse, Female)

Similarly, a gynecology doctor explained how her conflicting values led her to stop doing social termination of pregnancy:

I have stopped doing social terminations of pregnancy because I found them too difficult…I found that I was struggling with it ethically and morally because, quite often, patients would come in and that would be their second or third termination […] and I just stopped because it felt wrong. (Claire, Doctor, Female)

Only a few participants opted for such a dramatic change as a result of experiencing identity conflict and throughout the interviews we found an answer to this. Healthcare professionals go through a long training that allows them to realize which specialty they feel more aligned with. This doctor explained clearly that healthcare professionals tend to choose the path that they know may cause them less conflict:

I guess, one of the reasons I would never be able to work in obs and gynae is because, I think, there would frequently be tensions in me because I believe most heartedly that termination is not ok…and it’s not something I can condone, it’s not something I could be involved in. (Jess, Doctor, Female)

However, since the differences between medical areas are numerous, some of them might not be detectable during training. Additionally, personal values can change over the course of one’s life, and what was acceptable at a young age might become unacceptable with maturity and critical life events. For instance, the doctor who stopped doing social termination of pregnancy noted:

I was always able to do it by looking at it more medically rather than looking at it from a life point of view…but now I see it more as a life than a very early embryo, a bunch of cells […] I suppose earlier in my career, I probably thought ‘oh, it’s only a bunch of cells, it’s not a life, it’s not a baby, it’s just an early embryo’ […] I think the change started when I was trying to get pregnant and I had a miscarriage […] so now I can’t do it anymore. (Claire, Doctor, Female)

Hence, the healthcare professionals tackled conflicts in their practice with a series of behavioral responses that could allow them to cope promptly or subsequently with such struggles. The participants also spoke unsolicitedly of the psychological consequences of the identity conflict. Since the insights they provided were of great interest to this study, we report in the following section our interpretation of the participants’ psychological responses to the perception and experience of conflicting values and identities.

#### Psychological Consequences of Identity Conflict

The participants spoke about identity conflict as having positive psychological consequences on their self. These consequences were reached through a long process that unfolded over time, leading to a personal development of learning and gaining life experience. As noted by this nurse:

It [conflict] changed my practice in the sense that my career changed direction, but I don’t think it affected it negatively […] I suppose, if there was any impact it was positive and that was good, I wouldn’t change anything […] so, I would say that it has definitely had a positive impact on me. (Lauren, Nurse, Female)

Also, the fact that the participants could gain real life experience through facing personal conflicts was seen as beneficial for their clinical and medical practice:

Dealing with those kinds of conflicts has always, and will, put me in a better position next time…so, even if you don’t get exactly the same situation, but similar situations, I will be able to manage that conflict […] a lot of it will always affect your future clinical practice…it adds to your experience, doesn’t it? (Laura, Nurse, Female)

Strictly related to gaining life experience, the learnt outcomes equipped and enriched the healthcare professionals with knowledge that could then be applied to other future critical cases. Indeed, by taking the ‘lesson learnt’ on board and holding on to that experience for future circumstances, the participants acknowledged that:

Everything teaches us every day…we’re all in a sort of learning journey and it might not be even something like “I’ve learnt this fact and this fact,” sometimes it’s more nuanced than that…it’s just something in that kind of situation taught me something, even if it’s not a fact. (Karen, Doctor, Female)

Hence, perceiving identity conflict seems to start a virtuous process of personal development and change, based on the accumulation of life experience and beneficial learning outcomes. Additionally, the experience of identity conflict stimulated the participants’ sense of personal growth and increased maturity and self-awareness:

When you experience dilemmas, you change…inevitably…It just doesn’t happen overnight and I think it’s the same with any kind of conflict or struggle you face…you grow with your role, with your knowledge, with your experience…it’s maturing really […] and also I think it’s about feeling more comfortable in your own skin and in your own belief. (Maddy, Doctor, Female)

Overall, time played a pivotal role in allowing the participants to develop a positive perception of the conflicts they experienced thanks to the processes of learning and personal growth. These processes prompted a sense of gaining knowledge, confidence and maturity that the participants treasured and made use of to tackle ethically charged cases in their practice.

## Discussion

### A Multilevel Model of Identity Conflict

**FIGURE 2:**
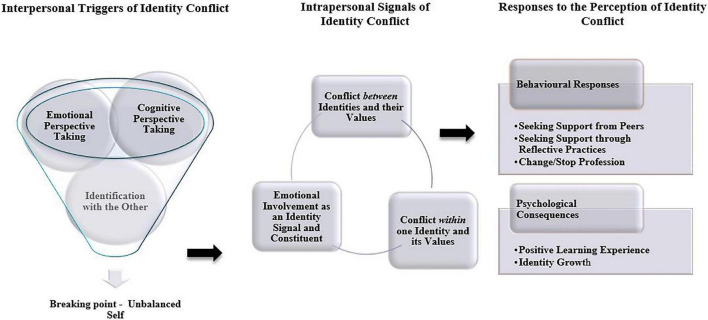
Illustration of findings.

The findings of our empirical analysis are depicted in Figure 2. Our results suggest that identity conflict can arise due to interpersonal micro processes, namely cognitive and emotional perspective taking and identification with the other. These micro processes can trigger latent, contrasting values and emotions to which individuals respond by actively seeking support from peers or through reflective practices, and rarely leaving or changing their profession. The overall experience of conflicting identity values has positive consequences on the participants, ranging from learning and gaining knowledge, to maturing and feeling personal growth. In the following two sections we explain how the findings relate to both our research questions and contribute to current literature, respectively.

### Healthcare Professionals’ Perception of Identity Conflict

In the first research question, we sought to respond to how identity conflict in interpersonal interactions is perceived and experienced at a deeper level in ethically charged situations. Building on a relational conceptualization of self, and consistent with the principle that our social relationships inform our self (Brewer and Gardner, 1996; Islam, 2020), the first contribution of this article to identity dynamics literature is the development and inclusion of interpersonal processes as potential triggers of identity conflict. Our findings show that identity conflict perception is strongly associated with cognitive and emotional perspective-taking processes, which affect the activation of professional and personal identities, adding to the inner notions of salience and centrality of more traditional identity theory approaches (Stryker and Serpe, 1982; Mitchell and Boyle, 2015). Hence, our study draws on, and merges, well-established intrapersonal dynamics with less explored interpersonal processes and unpacks identity conflict throughout its multilevel dimensions (Horton et al., 2014; Islam, 2020; Carminati and Gao Héliot, 2021). Identity construction has a strong relational component in professionals that makes them accountable to others and subject to various social controls (Ramarajan and Reid, 2020). Understanding their patient’s perspective enables healthcare professionals to establish a strong cognitive and emotional connection with them (Calvard et al., 2021). However, such a connection, a vital principle of the healthcare professional-patient relationship (Diamond-Brown, 2016), can function as a double-edged sword and challenge the distinction between the identities constituting their “selves” (Harding et al., 2014). Indeed, perspective taking initiates the third micro process identified herein, i.e., identification with the other. This process can activate shared and potentially less salient personal identities and values. In this sense, interpersonal relationships blur the boundaries between the self and the others (Sluss and Ashforth, 2007; Islam, 2020). Given the emotional and cognitive dimensions associated with perspective taking processes (Williams, 2012; Calvard et al., 2021) and identification with the other (Sluss and Ashforth, 2007), we believe that interpersonal interactions can give a meaningful explanation to identity conflict dynamics by representing one of the initial conditions generating those disharmonious feelings and values activated in the conflict.

Supported by virtue ethics insights and their focus on individuals’ moral characters rather than actions (Uhlmann and Zhu, 2014; Uhlmann et al., 2015), our second contribution to recent strands of identity theories is an in-depth understanding of the perception of identity conflict dynamics that goes beyond a misalignment between identities (Ramarajan and Reid, 2013; Ramarajan, 2014) and includes a tension between the values constituting those identities. More specifically, we extend identity conflict literature by focusing on the multiplicity of the values and imperatives involved in identity conflict and how they can influence professionals’ decision making. Research has shown how values, driving forces of identity formation, can spur individuals’ self-expression, self-verification, consistency and authenticity (Wright et al., 2017). By considering identities as multifaceted and polyhydric constructs, rather than monolithic entities, we bring this forward to now include the role of multiple values interacting in identity conflict dynamics. This interaction can shape the degree of (in)congruence between individuals’ multiple identities (Héliot et al., 2020), as well as influence professionals’ judgments and subsequent actions (Huhtala et al., 2020). Indeed, we find that identity conflict not only can happen between values belonging to different identities, but also between values that are associated with one, same identity, i.e., the participants’ professional identity. This is because the same professional values have the potential of being interpreted differently (Wright et al., 2017) due to the presence of other personal and intrinsically moral values that are triggered by the interpersonal processes at stake. Thus, illuminated by the focus on values, our study offers a deeper understanding of the sources of intrapsychic conflicts that professionals may experience in ethically charged situations.

In this regard, some participants reported identity conflict due to related moral values associated with their professional identity. Nielsen (2016) underlined that moral agency issues are part of the work of professionals and practitioners since they need to choose continuously between different principles and rules at work. In this sense, individuals seek for practical wisdom to define their identities appropriately as moral agents (Huhtala et al., 2020). This is in line with recent research on phronetic identity (Bardon et al., 2017), which is a “narrative in which an individual describes him or herself as questing for the wisdom to make appropriate decisions in ambiguous and equivocal situations, driven by the desire to do what is (notionally) right and good” (Bardon et al., 2017, p. 958). Phronetic identity is always *in-fieri*, in the process of becoming, since the quest of the ‘right’ way of doing things is informed and driven by a strong rational and emotional attachment to people (Bardon et al., 2017; Ramarajan and Reid, 2020). The participants in our study establish a strong attachment with their patients. By showing that the conflict they experienced can be interpreted as the unfolding and becoming of their phronetic identity through their professional and personal values, we thus offer fresh insights into how professionals perceive the interplay between moral values, identity conflict and ethical decision making.

Lastly, we contribute to the literature on identity dynamics by unraveling how emotions can act as a warning signal of an emerging identity conflict and a plausible way out of it. Research so far has shown that emotions are involved in the experience of identity-threatening situations (Cascón-Pereira and Hallier, 2012; Croft et al., 2015; Winkler, 2018) and have an agentic role in identity meaning construction and development (Ahuja et al., 2019). Extending this further, our study shows that emotions are reciprocally interlaced with the cognitive work of sense-making and sense-giving through which individuals try to make sense of ambiguous or unexpected events (Heaphy, 2017; Caza et al., 2018). Indeed, participants’ emotions can facilitate cognitive processes through their connection with values. Thus, through values, emotions are able to stir individuals to pursue the *right* course of action. Here, the emotions felt by participants are intrinsically of a moral nature since they motivate individuals to take action that could benefit others or the social order (Haidt, 2003; Huhtala et al., 2020). Hence, emotions function as a moral signal and a cue for individuals to act upon those identity values that were activated in the conflict. Building on studies reporting how felt emotions can influence individuals’ interpretation and action orientation (Heaphy, 2017), we also see emotions as reflexive processes that can help individuals to look back to gain that self-consistency they aim to achieve, but which is temporarily lost in the identity conflict. Thus, through reflexivity, individuals can better understand and manage their emotions ultimately enhancing their emotional intelligence (Stanley, 2021), which can foster better decision making, lower exhaustion and stress, as well as help in delivering high-standard quality of care (Martin et al., 2015; Szczygiel and Mikolajczak, 2018). Therefore, our findings show how skilled professionals attempt to calibrate the emotions involved in their experience of identity conflict, so as to engage in sense-making and sense-giving, and, subsequently, act morally following those identity values they deemed more appropriate in that situation.

### Healthcare Professionals’ Responses to Identity Conflict

Our second research question contributes to identity conflict literature by identifying some of the responses that individuals implement when experiencing conflicting identity values in ethically charged circumstances. Our findings thus extend current theoretical research on identity dynamics (see: Petriglieri, 2011; Ramarajan and Reid, 2013) by outlining unexplored behavioral responses and novel psychological outcomes as of identity conflict consequences following interpersonal interactions.

Firstly, even though the medical literature alludes to the healthcare profession as a job characterized by autonomy and independence (Hurst et al., 2005), our findings show that the participants rely greatly on their peers for support in challenging situations. Indeed, support stemming from expert peers, compared to managers or other authorities, is more likely to influence critical decisions (Mitchell and Boyle, 2015) and favor self-verification, needed to affirm one’s identity (Stryker and Serpe, 1982; Tajfel and Turner, 1986). Additionally, research has shown that peers’ help is critical in processing individuals’ own emotions and developing empathic accounts (Heaphy, 2017; de Lange et al., 2020). The approval of the medical community also functions as a way whereby healthcare professionals can strengthen their values and try to solve potential identity conflict (Wright et al., 2017). Hence, seeking support from peers can help in coming to terms with the conflicting values and emotions causing struggles and dilemmas in professionals. We thus suggest that peer support, as an element of the context that can influence intrapsychic processes (Islam, 2020), can be considered as a buffering condition ameliorating the negative outcomes ascertained by the literature in relation to identity conflict.

Secondly, our findings highlight the importance of undertaking reflective practices for healthcare professionals. Individuals in complex ever-changing organizations are constantly involved in identity conflict since they deal with critical challenges and the existential worries that accompany them (Caza et al., 2018). Making a purposive effort to devote time and thoughts to reflect on previous challenging experiences can help to improve decision-making skills, thanks to a virtuous learning cycle (de Graaf, 2019). Reflexivity is thus produced through processes of experiential learning whereby values can be maintained by reflective professionals and practitioners (Wright et al., 2017). Hence, responses to identity conflict can also be developmental (Ramarajan and Reid, 2020), since the voluntary enactment of this ‘reflection-on-action’ can facilitate and stimulate learning in individuals by developing abilities to draw analogies and comparisons between different critical cases (de Graaf, 2019). Such abilities may then lead individuals to learn about themselves and think more flexibly about their personal struggles (Ramarajan and Reid, 2020), thus favoring the resolution of the identity conflict experienced at the workplace. Therefore, undertaking reflective practices can help healthcare professionals to lessen the discomfort caused by conflicting values.

Our study also offers empirical support for a recent perspective on identity conflict that underlines its positive influence on individuals in the long run and its relationship with identity change (Horton et al., 2014). Events can foster momentum in individual’s trajectory, nudge its direction and serve as tipping points for radical changes (Ashforth and Schinoff, 2016). Traditionally, empirical identity conflict research has been mostly cross-sectional and has associated the experience of identity conflict with negative psychological outcomes (Brook et al., 2008; Karelaia and Guillén, 2014). Nonetheless, our findings highlight that positive identity growth is not only perceived as a long-run consequence of identity conflict, but also as a sense of maturity and confidence built on facing such a conflict. Recent research conducted in the NHS on the influence of religion in workplaces has indeed underlined that in some cases, identity conflict can positively contribute to personal thriving (Héliot, 2022). Hence, delineating this growth allows us to add a novel temporal dimension to individuals’ identity conflict, able to address that the way individuals react to identity conflict is grounded in their past and present experiences and can positively shape their future personal self-perception and self-development.

## Practical Implications

Our study reveals that identity conflict in ethically charged situations is not only triggered by intrapersonal dynamics but also interpersonal interactions. Viewing identity conflict through this multilevel dimension allows us to bring fresh insights into understanding how people can cope promptly with identity conflict. Our study encourages policy makers and HR to offer prevention plans and create a psychologically safe work environment (Héliot et al., 2020). It also spurs professionals to be more aware and mindful of the values and the emotions experienced in ethically charged situations. Since both values and emotions may have a moral side, they could trigger and signal identity conflict in interpersonal interactions and might pose a threat to professionals’ psychological and behavioral outcomes during their daily practices. Those professionals who have high chances of experiencing identity conflict must discover who they want to be and how they want to act in order to establish not only an internal consistency, but also a genuine connection with their patients and be effective in their practice. By doing so, they would also be able to pursue a *right* course of action and be true to themselves.

## Limitations and Future Research

Like all research, our work has limitations that need to be noted. Firstly, our study was only conducted across English NHS Trusts. Although this healthcare context provides an optimal setting for investigating with transparency the processes we were interested in, its peculiarity, country specificity, ethnicity and cultural background as well as extreme nature may limit the generalizability of our findings. Therefore, we encourage exploring other ethically charged cases that could stimulate identity conflict in different professional contexts. Similarly, although healthcare can be considered as a prototypical profession (Strauss, 1975; Pratt et al., 2006), studying other professionals, e.g., lawyers, would be helpful to assess whether the processes and dynamics found herein could also occur there. Secondly, at the time of the data collection, COVID-19 pandemic had not stricken, yet. Since we acknowledge that some results, such as peer social support, could be different had the research been conducted during or after the pandemic, we call for future studies to explore how our findings could be now different due to the pandemic. Lastly, considering that people bring in and leave out different levels of depths of themselves at their workplaces, we recommend exploring a complementary phenomenon of identity conflict, namely identity enhancement (Ramarajan et al., 2017), to unravel how multiple identities and values can enhance each other and how individuals manage the delicate balance with identity conflict.

## Conclusion

Due to the complexity and diversity of today’s society and organizations, professionals are more exposed to face identity conflict in their workplaces. In this article, we proposed a multilevel model of identity conflict by exploring its interpersonal and intrapsychic dynamics, together with its behavioral and psychological consequences on individuals. Illuminated by the critical case of healthcare professionals in ethically charged situations, such multilevel exploration allowed us to grasp the fine equilibrium between the self and the surrounding environment. In so doing, we were also able to unravel how values and emotions are interlinked to identity conflict, as well as how peer support and identity growth represent crucial responses to identity conflict in ethically charged circumstances. Since we believe that similar identity conflict dynamics can occur in several other professions and organizational contexts, we call for quantitative research to further investigate the processes and the mechanisms suggested in this model in professionals facing ethically charged situations.

## Data Availability Statement

The datasets presented in this article are not readily available because of the sensitive topics discussed and the reference to healthcare organizations. Requests to access the datasets should be directed to LC, l.carminati@utwente.nl.

## Ethics Statement

The studies involving human participants were reviewed and approved by University of Surrey Ethics Committee. The patients/participants provided their written informed consent to participate in this study.

## Author Contributions

Both authors listed have made a substantial, direct, and intellectual contribution to the work, and approved it for publication.

## Conflict of Interest

The authors declare that the research was conducted in the absence of any commercial or financial relationships that could be construed as a potential conflict of interest.

## Publisher’s Note

All claims expressed in this article are solely those of the authors and do not necessarily represent those of their affiliated organizations, or those of the publisher, the editors and the reviewers. Any product that may be evaluated in this article, or claim that may be made by its manufacturer, is not guaranteed or endorsed by the publisher.

## Footnotes

1 Examples of ethically charged situations are: resuscitation status, withholding and withdrawing medical therapies, palliative care, coma, vegetative state, and brain death.
