# Supplementary material for: Between Multiple Identities and Values: Professionals’ Identity Conflicts in Ethically Charged Situations
Source: Front Psychol. 2022 Apr 20;13:813835. doi: 10.3389/fpsyg.2022.813835 (PMC9068603; doi:10.3389/fpsyg.2022.813835)
Supplement: Supplementary file 1 [file Data_Sheet_1.PDF]

**Appendix A**

**Selected Questions from the Interview Protocol**

1. What influenced you to become a doctor/nurse?
2. In your eyes, what are the values of a typical doctor/nurse?
3. Besides the values related to being a doctor/nurse, do you bring any personal values with you when you come to work?
4. Are there any occasions when your professional values ask too much of you as a person?
5. Can you recall a personal struggle, a dilemma, you experienced in your clinical or medical practice?
6. Why did you perceive it as an ethical dilemma?
7. What personal values do you think were involved when you experienced an ethical dilemma?
8. Could you briefly describe the decisions that you made and what you did when you perceived the struggle?
9. Please take your time to think of other reasons or factors that influenced your decision in those situations?
10. Now, could I briefly bring you back to Baby Charlie Gard's case at the GOSH hospital in 2017? This baby was born with a very severe genetic disease and the hospital originally said that there was a very expensive, experimental treatment from the States that could be used to try to save the baby. The parents involved the media to collect the money for that, but by the time the money was collected, the hospital then said the treatment was futile. Pope Francis and some Italian hospitals were involved in the discussion, Donald Trump as well but, in the end, the Supreme Court said that the hospital was right and the treatment was futile, and the baby died in palliative care. So, can I ask you what your thoughts are regarding this case?

Note: Question 10 about Baby Charlie's case was introduced after the pilot interviews when it became clear to the authors that some healthcare professionals struggled and found hard to open up about their own personal ethically-charged experiences. Hence, Baby Charlie's case, a popular case in the UK that divided the public opinion for months, was used as a sort of "warm-up" question to help participants to feel more at ease and not judged in their moral opinion.

## Appendix B

## Table 1: Research Question 1: Exemplary Quotations for First-Order Themes

| Exemplary Quotations | Description | First-order Themes |
|---|---|---|
| "So, I think it probably was the one where I was in charge of my friend, which was "**what do you want for yourself?", because that's what you can relate to, isn't it? But what you want for yourself might not be what anybody else wants for themselves, and so it's that**…that is when you probably **need to try to put things into boxes a little bit** and you need to try to explore what the patient would want, what the relative would want and also what other qualified people would do, what would be accepted management in that situation…" (David, Doctor, Male)<br><br>"I think that sometimes **when we are faced by challenging situations and we try to support people, we tend to reflect on what we value the most** […] but those are my values and my way of thinking…**so, when we are looking for ways of supporting people, we often think about what could help ourselves, and that could be quite misleading, meaning that, in the worst case scenario, it could actually be inappropriate, or that you would end up doing something that is just ineffective, so you wouldn't cause any harm, just want it to work**… " (Christian, Nurse, Male) | The first-order themes (1a, 1b and 1c) were developed to capture the way the participants were trying to understand their patients.<br><br>The desire to understand the other's perspective and feelings, putting themselves in the patient's shoes, were constant themes in the data.<br><br>This necessity to understand the other was, however, spurred by the sense of similarity/ "sameness" with the patient. The higher the identification with the patient, the higher the chance of experiencing inner conflict. | **1a. Trying to put/imagine yourself in someone else's shoes (cognitive perspective taking)** |
| "Because…**it's called empathy**…**I put myself in that person's position…it's something automatic, you know, I have children myself so I know how probably, sort of, those parents would think and feel**…it's terrible…and you would fight for everything for an only child…" (Simon, Doctor, Male)<br><br>"I suppose, yes**, I have empathy, as a daughter and as a mother, with patients** […] when I'm faced with similar situations in the hospital, when somebody who is similar to my dad definitely influences my thoughts about them and what I think should happen…and **I try to put myself in the shoes of their daughters, sons, wife and try to imagine what they would want because I know how they would feel**…generally, I think it fits, and I wouldn't change what I did…**I just think that it probably gives me more empathy with people**…" (Rianne, Nurse, Female) | | **1b. Empathic understanding of the situation and of others' feelings (emotional perspective taking)** |

| | | |
|---|---|---|
| “**I do think that it is very stressful when we have younger patients or patients who are going to die but have young children** …that is extremely stressful…**and I think that the young staff who identify with them, you know, they might be the same age as the mother, they might have children of the same age**…**and I felt that too, when there was a 62-year-old mother in her bed with her two sons and two daughters weeping at the bedside you of course immediately think “Oh, that could be me”**…and **when you can identify in a personal way, I think that's very hard**…a professional approach can help you to come to terms with that because it can help you to understand that family's scenario better…” (Andrea, Nurse, Female)<br><br>**“Unfortunately she died and it always hits a little bit more when the patient is around your age group, and that’s quite a difficult situation to deal with** […] I think it was a conflict for me because **it felt so close and personal**…” (Zoe, Nurse, Female) | | **1c. “Identification”/Sameness with the others** |
| “I think it is the big question about…**you know, if there’s a patient lying in the bed and their ventilator is removed and they die…are you allowed to think that was the right decision?...you know, ‘is there a right or wrong in that? Is there a truth in that?’**…**these are huge ethical questions and we, as moral people, are all different, with different perspectives** […] **I think the dilemma or the struggle is that ethical bit that says “actually, we are healthcare professionals, we can't do what the patient is asking**…because we can keep them pain free, we can sedate them, we can comfort them, we can support them, we can care for them…we can do all those things, **but we cannot do what they're really asking, which is to hasten the end of their life**…” (Clio, Nurse, Female)<br><br>“I used to do intensive care, ok? And it means the sickest patients in the hospital arrive there and they get better or they get worst, ok? And **there are two schools of thought**…**one is “let’s give everyone a chance”** pretty much despite the medical background, whether it is a 100-year-old lady who never leaves her house, on a wheelchair, or something like that**…“you should just treat her, and just try, she might get better, she has some sort of quality of life at home, just give her a chance, she might get better”…the other school of thought is “she has no quality of life, she is dying pretty much, no heart function, we are just prolonging her death, pretty much”**…ok? **I think those two are opinions I struggle with**…” (John, Doctor, Male) | The 2a, 2b, 2c first-order themes were developed because the participants recognized that they could perceive struggles (→ “identity conflict”) in their medical practice between the different values they hold.<br><br>The realization and understanding of the inner conflict can happen through a sort of rationalization of the process and the values involved. This conflict can involve “professional vs professional” values or “professional vs personal”.<br><br>Sometimes this process of awareness of the conflict can also happen through a more emotive way, i.e., evaluating and making peace with the feelings and emotions at stake.<br><br>In any case, the participants strongly highlighted the importance of managing the values to navigate out of potential struggles. | **2a. Cognitive recognition of inner conflict *between* different identities (e.g., professional identity/values vs personal identity/values)** |
| “I think the trickiest thing I have ever had to do was about 2 years ago, when I was working in a different hospital, and **we had a patient who, apparently, was a pedophile…so, obviously, “how on Earth are you going to look after a patient like** | | **2b. Cognitive recognition of inner conflict *within* the same identity (e.g., professional values vs professional values)** |

| | | |
|---|---|---|
| **that?"…and that was really challenging, personally, because of all my personal beliefs and viewpoints about that, I just couldn't accept that**…" (Martha, Doctor, Female)<br><br>"**I think there are probably a few occasions…when it's difficult to marry what you are trying to do professionally with maybe what your own beliefs are** […] that's the biggest challenge sometimes […] **I found that incredibly hard, because I wanted to support her, and I didn't want her to feel like I was judging her, obviously, whereas inside I was thinking "no, no, no, this is not the right thing at all"**[…] **but I had to go with what her wishes were**…so, yea, that was incredibly difficult […] I suppose that my struggle was **in terms of personal values…because family is really really important to me, I've got a very close family and I just think that them not being aware of the situation, if it was my situation, would be impossible to imagine** …" (Daphne, Nurse, Female) | | |
| "Well, **it** [the experience of the conflict] **upsets me**…**I feel I'm not necessarily doing the right thing**…the right thing medically, but possibly doing the right thing for them as people […] you are just trying to do everything to help them, but **who knows if I'm actually doing that**…sometimes **this uncertainty is eating me inside** and, you know, I have to eat a box of chocolates because…it's a bit **like in Harry Potter, where Dementors suck out your soul, and you feel that your soul has been just sucked out…so, yes, chocolate, chocolate and friendship [colleagues]**…" (Birgit, Doctor, Female)<br><br>"It was just that **daunting feeling**, you know, "what happens if something goes wrong? What happens if this or this happens?"…you know, **all those kinds of situations or dilemmas you could potentially be faced with all seem sort of like possibilities I suppose**...and **it's not a nice feeling when you are out of your comfort zone**…" (Lisanne, Nurse, Female) | | **2c. Feelings (daunting and unsettling feelings) and emotions (fear, anxiety, scared) recognized when facing identity conflict** |

## Table 2: Research Question 2: Exemplary Quotations for First-Order Themes

| Exemplary Quotations | Description | First-order Themes |
|---|---|---|
| “**And of course you use your peers**, you know, there are other consultants in different university hospitals who I can email and say “I'm not sure what to do about this, you know”, so, again, **that’s where the team work comes in and fraternity…and that’s why you can’t work in isolation in oncology**…you would not cope emotionally…or I wouldn’t …” (Mary, Doctor, Female)<br><br>“I think that **ethically challenging decisions are made as part of multidisciplinary team**…and I think that sort of takes us back a little bit to **the importance of working within a team, even though we are working independently on the wards, the importance of having that team time**…” (Erwin, Nurse, Male) | These first-order themes (1a, 1b, 1c) were developed because the participants unanimously referred to the need to discuss personal struggles, uncertainty and doubts, with others (especially peers) as a response to the experience of identity conflict.<br><br>They also noted that, besides the support from their team, personal reflection was also important as a way to understand, cope with and rationalize the emotionally- and ethically-charged situation they had been in.<br><br>Additionally, they underlined that, as a sense of anticipation of potential future conflict in their clinical practice, they tended to choose medical paths that, although still within their vocation, were more aligned with what they defined as “their personality”.<br><br>The participants finally pointed out that, albeit in only two cases, the experience of inner conflict resulted in changing or stopping the medical practice. | **1a. Seeking support from peers when experiencing conflicts/dilemmas** |
| “**Nurses are very good at reflecting, it’s a huge part of our professional development…with the idea of “would I have done anything differently?”** […] **reflection is a learning cycle, kind of preparing yourself for similar things in the future**…” (Igor, Nurse, Male)<br><br>“I suppose as a doctor, as a healthcare professional, you are always learning from every case you are involved with…and **reflecting on that, and revaluating what you think is right or you would do differently next time**…” (Hannah, Doctor, Female) | | **1b. Seeking support through reflective practice (sometimes done as a team practice)** |
| “You hear the Catholic members who are very clear on their views on contraception…and, again, Catholic GPs are those who feel very strongly that all contraception is against God’s will so, you know, how do they do their job?…because so much of a GP’s case is contraception…if you refuse to be involved in any of that, you know, is that right for the patient?...emmm…so I guess, again, **I’ve seen people being actively dissuaded from going into general practice, or leaving it after few months, if they are very strong Catholics and believe that, because you can’t…they would say “you can’t do your job effectively if you are not willing to do this”**…” (Jess, Doctor, Female) | | **1c. Changing or stopping medical practice when experiencing or foreseeing potential (vocational adjustment) personal conflicts/struggles** |

| | | |
|---|---|---|
| "I suppose as a doctor, as a healthcare professional, **you are always learning from every difficult case you face**…and when there are lives involved, well, you always learn more…and this is good, because **you enrich yourself with life experiences that you could have never learnt during medical school** […] and revaluating what you think is right or you would do differently next time, that is empowering…" (Cass, Doctor, Female)<br><br>"I guess **we all learn day by day…I think that every time we approach something, especially the dilemmas, you cannot learn […] it is because, I guess, by definition they're challenging us**…**there is always something that can be learnt if you are open to it** […] sometimes it's difficult to grasp what you've learnt, but it's there and you know that next time you'll do a little bit better…**it's an endless learning cycle I guess**…" (Martha, Doctor, Female) | The 2a and 2b first-order themes were created because, quite surprisingly, the participants perceived inner conflict as, overall, a positive learning experience for their personal growth.<br><br>Both the doctors and nurses agreed that identity conflict was crucial for improving their knowledge and their clinical experience. In doing so, the conflict was seen as a learning process through which they could improve their medical practice. | **2a. Identity conflict as a positive life experience involving learning and gaining knowledge** |
| "I think there isn't a firm answer to that…**over the years you build those values, characteristics through experience, you do transform, you don't change, but you evolve as a doctor and the way you face dilemmas also evolves with you** […] **so you become more firm, more consistent,** your leadership coat is starting to build up and that **becomes part of your personality, who you are**…and I think that **gives you more confidence and affects, inevitably, how you look and face dilemmas**…" (Erik, Doctor, Male)<br><br>"**It definitely made me more confident, to acknowledge that I'm not confident in what I'm doing**…and I think it made me realize that it's ok for me, as a nurse lead, to actually not know something…**so I think for me, in a nutshell, it made me more open, more aware of my own limits…and in terms of my practice, well, I've always been like this**…" (Anne, Nurse, Female) | | **2b. Identity conflict as a way of growing in one's role (identity growth) and becoming more confident and stronger** |
